# Experimental data on glucose and energy levels of orange mud crab, *Scylla olivacea* at different water velocities

**DOI:** 10.1016/j.dib.2020.105232

**Published:** 2020-02-04

**Authors:** Muhammad Taufik, Adnan Amin-Safwan, Abdul Rahman Mohd Nordin, Ismail Shahrul, Ambok Bolong Abol-Munafi, Mhd Ikhwanuddin

**Affiliations:** a Institute of Tropical Aquaculture and Fisheries, Universiti Malaysia Terengganu, 21030, Kuala Nerus, Terengganu, Malaysia; b Faculty of Informatics & Computing, Universiti Sultan Zainal Abidin, Besut Campus, 22200, Besut, Terengganu, Malaysia; c Faculty of Ocean Engineering, Technology and Informatics, Universiti Malaysia Terengganu, 21030, Kuala Nerus, Terengganu, Malaysia; d STU-UMT Joint Shellfish Research Laboratory, Shantou University, 515063, Guangdong, China

**Keywords:** Aquaculture, Crustacean, Hemolymph, Hydrodynamic, Environmental stressor, Energy distribution, Water turbulence

## Abstract

The present datasets were conducted to investigate glucose concentration in hemolymph, energy levels at selected body parts (hepatopancreas, muscle, gonad), and feces among different sexes of crabs cultured at four different water velocities (0, 20, 40, and 60 cm/s) during a 60-day culture period. A total of 102 immature crabs (51 males, and 51 females) were sampled from Kuala Muda, Kedah coastal water, Peninsular Malaysia (5°39′N 100°19′E) from April to November of 2018. Results indicated that glucose concentration was the highest at water velocity of 60 cm/s for both male and female crabs (♂: 3.76 ± 0.08 mmol/L; ♀: 3.63 ± 0.06 mmol/L), whereas at 0 cm/s, the lowest levels of glucose concentration (♂: 0.13 ± 0.08 mmol/L; ♀: 0.19 ± 0.06 mmol/L) were recorded. As for energy analysis in hepatopancreas, results showed that both male and female crabs recorded the highest levels at 0 cm/s (no flow) with 37.919 ± 0.07 KJ/g and 34.636 ± 0.50 KJ/g, respectively. Energy for locomotion (muscle) of male crabs recorded the highest at 0 cm/s (♂: 26.823 ± 0.06 KJ/g), meanwhile for females, the highest was recorded at 20 cm/s (26.607 ± 0.34 KJ/g). Energy for reproduction of males could not be compared due to an insufficient available amount of testes/vas deferens, whereas female crabs recorded the highest energy usage at 20 cm/s water velocity (♀: 37.895 ± 0.08 KJ/g). For feces, both male and female crabs recorded the lowest energy at 60 cm/s (♂: 5.841 ± 0.03 KJ/g; ♀: 5.393 ± 0.01 KJ/g). Glucose assessment showed a direct relationship between increased velocity and glucose secretion in hemolymph at high velocity of 60 cm/s (stress condition) compared to other treatments. Regarding energy analysis, this research improved the mechanism of hepatopancreas, gonad, muscle and feces functions in development and reproduction, while it shed light on the influence of velocity on energy metabolism of *S. olivacea.*

**Table:** Specifications Table

Subject	Agriculture and Physiology; Aquaculture
Specific subject area	Physiology; Fundamental Biology; Energy allocation; Stress factor
Type of data	Tables
How data were acquired	Sampling activities, hatchery work, sample dissection and laboratory analysis. The crabs were cultured in a novel flow test simulator design Re-circulating Marine Aquaculture System (RMAS). Glucose concentration was measured using a SIGMA kit (Sigma-Aldrich, USA) and energy production was measured by using Bomb Calorimeter C 2000 (IKA, USA)
Data format	Raw, filtered and analyzed
Parameters for data collection	Four different levels of water velocity (0, 20, 40 and 60 cm/s) were tested on mud crab. Every 15 days, throughout a 60-day culture period (Day 0, 15, 30, 45 and 60), three crabs were selected from each treatment for assessment
Description of data collection	Investigation of 102 immature crabs (51 pairs of male and female) by sampling of hemolymph, specific tissues (hepatopancreas, gonad, and muscle), and feces for measurement of energy. The hemolymph from third walking leg was withdrawn by using a 1 cc/ml (TERUMO) syringe for glucose analysis, whereas the tissues were freeze-dried in preparation for proper sample burning
Data source location	Kuala Muda, Kedah coastal water, Peninsular Malaysian (5°39′N 100°19′E) and Crustacean Hatchery of Institute of Tropical Aquaculture and Fisheries (AKUATROP), Universiti Malaysia Terengganu (UMT)
Data accessibility	Data was provided in this article
Related research article	M. Taufik, M. Hidayah, I. Shahrul, A.R. Mohd Nordin, A.B. Abol-Munafi, M. Ikhwanuddin. Locomotor, escaping activities and fatty acids composition of mud crab, *Scylla olivacea* at different water velocities, J. Teknologi (Sci. Eng.) 82 (1) (2020) 9–18. https://doi.org/10.11113/jt.v82.13861 [1]

**Table:** 

**Value of the Data** • Knowledge related to the effect of water velocity on physiological stress can serve as a guideline for the optimal velocity necessary during grow-out phase in captivity [2]. • Crab aquaculturist and crab farmers are benefits on this data since it can be used during the fattening activity of the immature crabs. • Investigation of crab hemolymph and tissue is crucial to understanding the ecosystem's impact on the general functioning of a crab through velocity tolerance. • Experimental data on ecological energy could be further useful to predict several ecological hypotheses, such as constructing relationships between different velocities and offspring or reproductive ability, explaining predator conduct foraging and confirming organism physiological status [3].

## 1 Data

**Fig. 1:**
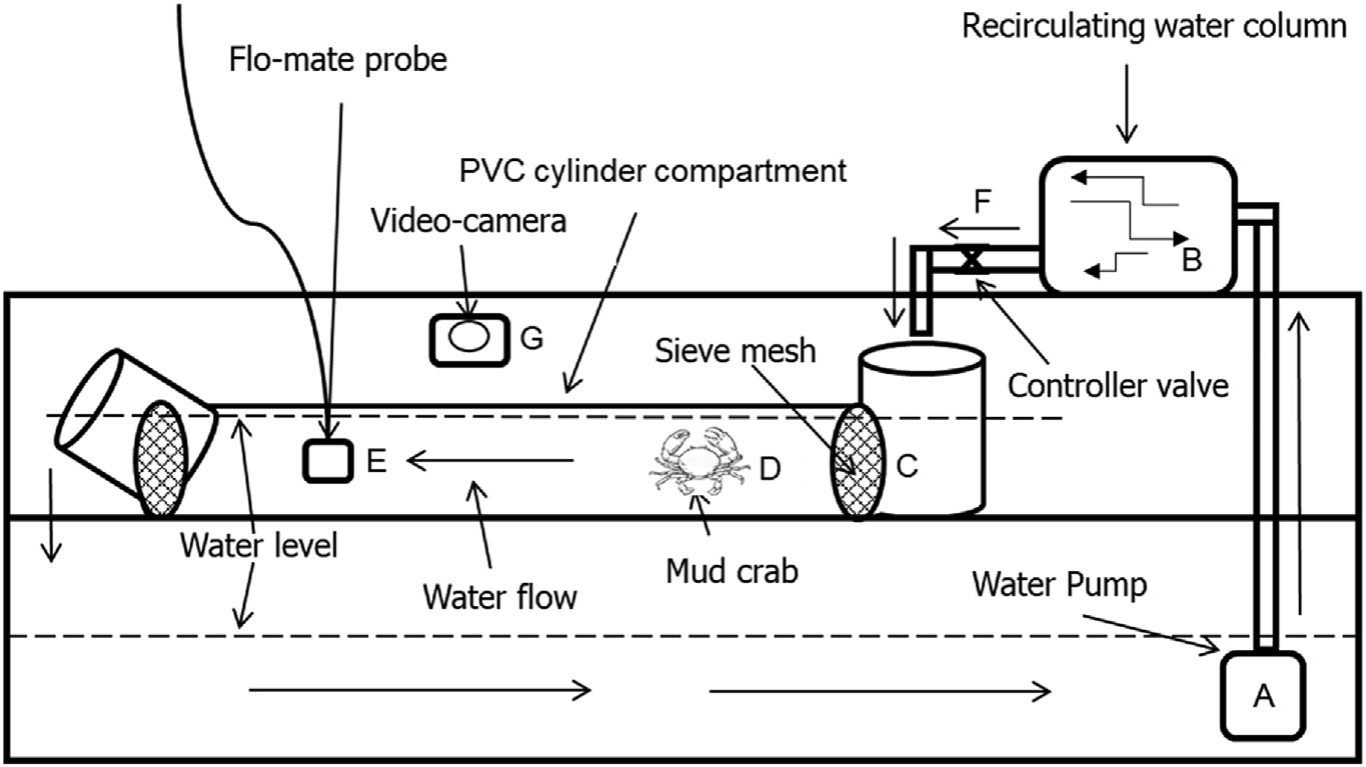
PVC pipe model for velocity treatments practiced during present experiment. (A) Water pump, (B) Recirculating water column (act as filter), (C) Sieve mesh (to prevent crab from escaping), (D) Mud crabs (as samples), (E) Flo-mate probe (used for measuring velocities), (F) Controller valve (used for adjusting velocities), (G) Video-camera (for recording crab behaviour).

Included in this article are the raw data and descriptive data (means) on the effects of water velocities on glucose and energy levels of the orange mud crab, *Scylla olivacea*. The shared data are recordings from various works including; sampling activities (mud crab), hatchery phase (culturing period for water velocity treatments – Fig. 1), and laboratory work, involving glucose level determination in the hemolymph (Table 1), energy reserves in hepatopancreas (Table 2), energy for locomotion in the muscles (Table 3), energy in the gonad for reproduction (Table 4), and energy usage in the feces (Table 5) of *S. olivacea* cultured for 60-days.

**Table 1:** Raw data on glucose levels (mmol/L) between sexes of *Scylla olivacea* at different water velocities (0, 20, 40, and 60 cm/s) sampled every 15 days within a 60-day culture period (n = 3).

	Days	0			15			30			45			60		
	Treatment	R1	R2	R3	R1	R2	R3	R1	R2	R3	R1	R2	R3	R1	R2	R3
Male	0 cm/s	0.22	0.11	0.06	0.22	0.44	0.28	0.83	0.56	1.17	1.94	1.72	1.83	1.22	1.50	1.72
	20 cm/s	0.22	0.11	0.06	0.06	0.22	0.22	0.44	0.94	0.44	1.83	1.67	1.78	0.73	0.99	0.86
	40 cm/s	0.22	0.11	0.06	0.06	0.44	0.22	1.39	1.22	1.33	3.28	3.33	3.06	3.06	2.94	3.00
	60 cm/s	0.22	0.11	0.06	0.50	0.28	0.50	2.17	1.56	1.56	3.72	3.67	3.67	3.78	3.67	3.83
Female	0 cm/s	0.22	0.22	0.11	0.67	0.50	0.39	0.17	0.13	0.28	0.28	0.50	0.28	0.06	0.17	0.22
	20 cm/s	0.22	0.22	0.11	0.72	0.72	0.61	0.64	0.77	0.79	0.72	0.61	0.72	1.98	2.06	1.14
	40 cm/s	0.22	0.22	0.11	1.89	1.83	2.17	2.50	2.67	2.61	3.06	2.78	2.89	3.06	2.83	3.22
	60 cm/s	0.22	0.22	0.11	3.06	2.72	2.83	3.17	3.39	3.33	3.44	3.33	3.83	3.67	3.56	3.67

**Table 2:** Raw data on energy reserves (KJ/g) in hepatopancreas between sexes of *Scylla olivacea* at different water velocities (0, 20, 40, and 60 cm/s) sampled every 15 days within a 60-day culture period (n = 3).

	Days	0			15			30			45			60		
	Treatment	R1	R2	R3	R1	R2	R3	R1	R2	R3	R1	R2	R3	R1	R2	R3
Male	0 cm/s	26.39	26.98	26.82	30.00	30.99	30.66	34.39	34.93	34.85	35.09	35.03	35.98	37.98	37.84	37.93
	20 cm/s	26.88	26.22	26.87	28.88	28.78	28.92	31.89	31.29	31.30	26.05	26.99	26.84	30.87	30.83	30.39
	40 cm/s	26.86	26.77	26.87	26.31	26.42	26.52	30.85	30.98	30.21	23.83	23.43	23.46	25.90	25.74	25.81
	60 cm/s	26.81	26.58	26.71	26.78	26.88	26.54	31.11	31.84	31.98	24.65	24.99	24.58	22.98	22.73	22.73
Female	0 cm/s	28.05	28.13	28.44	31.62	31.89	31.88	34.07	34.99	34.86	33.93	33.08	33.82	30.91	30.87	30.80
	20 cm/s	29.00	28.78	28.76	32.89	32.97	32.54	33.10	33.80	33.87	30.90	30.85	30.91	29.80	29.73	29.70
	40 cm/s	28.92	28.74	28.76	30.22	30.88	30.22	34.31	34.79	34.97	31.35	31.85	31.69	29.68	29.08	29.67
	60 cm/s	28.79	28.90	28.77	35.76	35.99	35.44	30.87	30.92	30.98	28.94	28.49	28.50	25.99	25.83	25.40

**Table 3:** Raw data on energy locomotion (KJ/g) in muscles between sexes of *S. olivacea* at different water velocities (0, 20, 40, and 60 cm/s) sampled every 15 days within a 60-day culture period (n = 3).

	Days	0			15			30			45			60		
	Treatment	R1	R2	R3	R1	R2	R3	R1	R2	R3	R1	R2	R3	R1	R2	R3
Male	0 cm/s	4.70	4.69	4.69	26.78	26.80	26.89	24.57	24.99	24.35	23.05	23.76	23.98	19.83	19.24	19.83
	20 cm/s	4.69	4.69	4.67	21.83	21.98	21.94	23.42	22.45	23.23	22.53	22.95	22.50	18.85	18.96	18.97
	40 cm/s	4.69	4.69	4.68	23.07	23.77	24.21	22.48	22.94	22.94	20.79	20.23	20.98	16.09	16.41	16.40
	60 cm/s	4.62	4.69	4.70	22.77	22.87	22.98	20.39	20.69	20.43	20.82	20.94	21.34	13.98	13.77	13.90
Female	0 cm/s	21.10	21.43	22.72	15.30	22.36	20.39	18.53	18.95	18.50	23.04	23.94	23.32	20.47	20.85	20.33
	20 cm/s	22.50	22.85	22.74	22.93	22.77	22.77	21.98	21.98	21.87	20.83	20.74	20.90	26.34	26.98	26.50
	40 cm/s	21.19	22.79	23.99	24.26	24.77	24.99	20.68	20.87	20.81	20.98	20.93	20.84	23.99	23.98	23.76
	60 cm/s	22.19	21.87	23.10	23.25	23.65	23.77	21.87	21.91	21.91	22.84	22.98	22.82	21.87	21.10	21.83

**Table 4:** Raw data on energy reproduction (KJ/g) in ovary of *Scylla olivacea* at different water velocities (0, 20, 40, and 60 cm/s) sampled every 15 days within a 60-day culture period (n = 3).

	Days	0			15			30			45			60		
	Treatment	R1	R2	R3	R1	R2	R3	R1	R2	R3	R1	R2	R3	R1	R2	R3
Female	0 cm/s	30.13	30.14	30.18	29.90	29.84	29.89	30.83	30.90	30.22	29.98	29.08	29.84	25.63	25.87	25.83
	20 cm/s	30.49	30.88	30.99	37.94	37.80	37.94	35.91	35.90	35.83	34.90	34.81	34.98	30.48	30.45	30.32
	40 cm/s	30.98	30.29	30.86	35.98	35.30	35.98	32.98	32.83	32.84	30.98	30.83	30.74	29.83	29.84	29.83
	60 cm/s	30.92	30.29	30.76	29.08	29.87	29.13	28.82	28.91	28.71	27.81	27.58	27.61	28.79	28.69	28.79

^∗^Male: Energy for reproduction for male cannot be compared since small amount of gonad present (insufficient sample to do an analysis).

**Table 5:** Raw data on energy usage in feces between sexes of *Scylla olivacea* at different water velocities (0, 20, 40, and 60 cm/s) sampled every 15 days within a 60-day culture period (n = 3).

	Days	15			30			45			60		
	Treatment	R1	R2	R3	R1	R2	R3	R1	R2	R3	R1	R2	R3
Male	0 cm/s	16.70	16.83	16.42	9.05	9.13	9.07	17.27	17.39	17.51	11.93	11.74	11.95
	20 cm/s	9.47	6.81	7.86	5.98	6.07	5.95	9.08	9.19	9.30	11.40	11.93	11.31
	40 cm/s	9.34	9.39	9.32	7.32	7.69	7.85	6.83	6.89	6.90	8.74	8.71	8.79
	60 cm/s	5.82	5.87	5.83	9.77	9.76	9.79	5.90	5.83	5.92	7.83	7.89	7.84
Female	0 cm/s	10.83	10.98	10.56	8.75	8.87	8.70	11.83	11.84	11.98	12.94	12.98	12.94
	20 cm/s	9.60	9.57	9.55	9.83	9.87	9.80	10.38	10.38	10.83	13.01	13.84	13.92
	40 cm/s	6.87	6.88	6.82	9.32	9.38	9.38	8.99	8.94	8.93	10.08	10.76	10.53
	60 cm/s	5.98	5.94	5.92	5.49	5.47	5.48	5.61	5.61	5.60	5.40	5.38	5.40

^∗^Day 0 not included since feces sample cannot be collected when crab newly cultured.

## 2 Experimental design, materials, and methods

### 2.1 Sampling and velocity treatments

A total of 102 immature crabs consisting of 51 pairs of male and female were sampled from Kuala Muda, Kedah coastal water, Peninsular Malaysia (5°39′N 100°19′E). The sampling methods and identification of crab maturity followed previous literature [[4], [5], [6]]. Sampled crabs were brought back to the Crustacean Hatchery at Institute of Tropical Aquaculture and Fisheries, Universiti Malaysia Terengganu for subsequent analysis.

Initial body weight (BW) and carapace width (CW) of each crab was measured and recorded based on previous methods [[7], [8], [9]]. In brief, BW was measured using a digital balance (accuracy: 0.01 g; Shimadzu model, Japan), while CW, using a six-inch liquid crystal display digital Vernier caliper (accuracy: 0.01 cm; Kingsmart brand, Hong Kong) by measuring the distance between the tips of the 9th anterolateral spine of the crab carapace [10,11]. Crabs were then held in an acclimatization tank under ambient light (500 lux) and temperature ± 26 °C for 1–2 days before being placed into the PVC pipe prototype. During the acclimatize stage and velocities experiments, the crabs were maintained in 20 ppt water salinity [5]. Cleaning activities included siphoning the feces (feces were collected for energy analysis), excess food sediment, and metabolic waste from the bottom of the tank every morning before introducing new chopped fish [12,13]. During acclimatization, lighting was eliminated and velocities were set to the required levels as crabs were placed in the PVC pipe setup for 24 h before starting Day 1 of the treatments. The closed system was allowed to run endlessly for 60 days. The water velocity treatments were adapted from Muhammad et al. [2] (Fig. 1). Each crab was maintained and fed with chopped scad fish, *Decapterus* sp. at 10% of the BW twice daily (0900 and 1700 h) during the 60-day cultured period [1,13]. Fifty percent of the water was changed every two days.

### 2.2 Glucose analysis

The hemolymph containing glucose was sampled and extracted (on Day 0, 15, 30, 45, and 60) from the third walking leg by using a sterile syringe with needle (1 cc/ml, TERUMO) [14]. The hemolymph was inserted into 1.5 ml centrifuge tubes and stored in −80 °C freezer until further analysis.

Samples were prepared as described in the manual of Glucose kit (Sigma-Aldrich, Inc., USA). Upon extraction, hemolymph samples were thawed and centrifuged at 15,000 rpm, 4 °C for 15 minutes. Next, the supernatant was removed and 100 μl of sample was mixed in 400 μl of deionized water, and further vortexed (15,000 rpm, 4 °C for 15 minutes). Approximately, 50 μl of hemolymph, and 100 μl of glucose assay reagent was pipetted into plate. The plate was then shaken gently and incubated at room temperature for 30 minutes to ensure uniform color throughout each well. The plate was read at 340 nm using a microplate photometer (Multiskan ™ FC, Thermo Fisher Scientific Inc.). The calculation for glucose levels determination followed Cheng et al. [15], and the final reading was averaged according to respective treatments.

### 2.3 Bomb calorimeter system procedure

The C 2000 calorimeter system is routinely used for determination of gross calorific values of solid and liquid substances. The following processes occur in the measuring cell during an experiment: The dry weight samples used were weighted approximately 1 g each. The samples were then compressed to become pellets using a compressor. Each pellet was then placed on ignition tread. The fuel sample (pellet) then enters the inner decomposition vessel of the measuring cell where pure oxygen flows inside through the oxygen filling apparatus until a pressure of 30 bar has been reached. Meanwhile, water from an external pressure source (water faucet, laboratory thermostat or cooler) flowed into the device and was heated to the working temperature (optionally 25 °C/30 °C). The inner vessel was filled with temperature-controlled water (at working temperature).

A stirrer ensured there was uniform distribution of heat in the water within the inner vessel as the water temperature of the outer vessel is controlled. The fuel sample (pellet) was then ignited electrically with the ignition wire using a cotton thread. As a result, the increase in temperature of the water in the inner vessel from combustion was measured and the gross calorific value was determined.

### 2.4 Statistical analyses

Differences between male and female glucose and energy levels were analyzed using one-way ANOVA and Tukey HSD test. Arrangement for the possibility of data distribution normality and standardization was assessed using standard normal plot and Cochran's C test. Any significance observed on glucose and energy level within velocities was tested using Kolmogorov-Smirnov test. Data on glucose and energy level was classified into *post hoc* categories through application of IBM Statistics Version 22 software and Microsoft Excel 2016.

## References

[1] Taufik M., Hidayah M., Shahrul I., Mohd Nordin A.R., Abol-Munafi A.B., Ikhwanuddin M. (2020). Locomotor, escaping activities and fatty acids composition of mud crab, *Scylla olivacea* at different water velocities. J. Teknol. (Sci. Eng.).

[2] Muhammad T., Ismail S., Ikhwanuddin M., Abol-Munafi A.B. (2019). Experimental data on behavioral, hepatosomatic, gonadosomatic indeces and total lipid of mud crab, *Scylla olivacea* at different velocity levels. Data Brief.

[3] Ciancio J., Suarez N., Yorio P. (2013). Energy density empirical predictor models for three coastal crab species in the southwestern Atlantic Ocean. J. Crustac Biol..

[4] Waiho K., Fazhan H., Ikhwanuddin M. (2016). Size distribution, length-weight relationship and size at the onset of sexual maturity of the orange mud crab, *Scylla olivacea*, in Malaysian waters. Mar. Biol. Res..

[5] Amin-Safwan A., Mardhiyyah M.P., Izzah-Syafiah M.A., Muhd-Farouk H., Manan H., Mahsol H.H., Nadirah M., Ikhwanuddin M. (2019). Dataset on reproductive status of ovary mud crab at different salinity levels. Data Brief.

[6] Ikhwanuddin M., Amin-Safwan A., Hasyima-Ismail N., Azra M.N. (2019). Dataset on body weight, carapace width increment and growth band count of mud crabs, *Scylla olivacea*.

[7] Liu C., Meng F., Tang X., Shi Y., Wang A., Gu Z., Pan Z. (2018). Comparison of nonvolatile taste active compounds of wild and cultured mud crab *Scylla paramamosain*. Fish. Sci..

[8] Duangprom S., Ampansri W., Suwansa-ard S., Chotwiwatthanakun C., Sobhon P., Kornthong N. (2018). Identification and expression of prostaglandin E synthase (PGES) gene in the central nervous system and ovary during ovarian maturation of the female mud crab*, Scylla olivacea*. Anim. Reprod. Sci..

[9] Song D., Shi B., Ding L., Jin M., Sun P., Jiao L., Zhou Q. (2019). Regulation of dietary phospholipids on growth performance, antioxidant activities, phospholipid metabolism and vitellogenesis in prereproductive phase of female swimming crabs, Portunus trituberculatus. Aquaculture.

[10] Ghazali A., Noordin N.M., Abol-Munafi A.B., Azra M.N., Ikhwanuddin M. (2017). Ovarian maturation stages of wild and captive mud crab, *Scylla olivacea* fed with two diets. Sains Malays..

[11] Amin-Safwan A., Muhd-Farouk H., Nadirah M., Ikhwanuddin M. (2016). Effect of water salinity on the external morphology of ovarian maturation stages of orange mud crab, *Scylla olivacea* (Herbst, 1796) in captivity. Pakistan J. Biol. Sci..

[12] Ikhwanuddin M., Azmie G., Nahar S.F., Wee W., Azra M.N., Abol-Munafi A.B. (2018). Testis maturation stages of mud crab (*Scylla olivacea*) broodstock on different diets. Sains Malays..

[13] Ikhwanuddin M., Mohamed S., Rahim A.I.A., Azra M.N., Jaaman S.A., Bolong A.M.A., Noordin N.M. (2015). Observations on the effect of natural diets on ovarian rematuration in blue swimming crab *Portunus pelagicus* (Linnaeus, 1758). Indian J. Fish..

[14] Amin-Safwan A., Muhd-Farouk H., Mardhiyyah M.P., Nadirah M., Ikhwanuddin M. (2018). Does water salinity affect the level of 17β-estradiol and ovarian physiology of orange mud crab, *Scylla olivacea* (Herbst, 1796) in captivity?. J. King Saud Univ. Sci..

[15] Cheng Q., Yu S., Myung N., Chen W. (2017). DNA-guided assembly of a five-component enzyme cascade for enhanced conversion of cellulose to gluconic acid and H_2_O_2_. J. Biotechnol..

